# An Incremental Voltage Difference Based Technique for Online State of Health Estimation of Li-ion Batteries

**DOI:** 10.1038/s41598-020-66424-9

**Published:** 2020-06-12

**Authors:** Arunava Naha, Seongho Han, Samarth Agarwal, Arijit Guha, Ashish Khandelwal, Piyush Tagade, Krishnan S. Hariharan, Subramanya Mayya Kolake, Jongmoon Yoon, Bookeun Oh

**Affiliations:** 1 Mobile Battery Research Lab, Samsung R&D Institute India-Bangalore (SRIB), #2870, Phoenix Building, Bagmane Constellation Business Park, Outer ring road, Doddanekundi circle, Marathahalli Post, Bangalore, 560037 India; 2 Advanced Battery Lab, Mobile Communication Business Division, SAMSUNG ELECTRONICS Co., Gyeonggi-do, 16677 Korea

**Keywords:** Chemical engineering, Computational science

## Abstract

Accurate state of health (SOH) estimation of rechargeable batteries is important for the safe and reliable operation of electric vehicles (EVs), smart phones, and other battery operated systems. We propose a novel method for accurate SOH estimation which does not necessarily need full charging data. Using only partial charging data during normal usage, 10 derived voltage values (${v}_{sei}$) are collected. The initial ${v}_{sei}$ point is fixed and then for every 1.5% increase in the Coulomb counting, other points are selected. The difference between the ${v}_{sei}$ values ($\Delta {v}_{sei}$) and the average temperature during the charging form the feature vector at different SOH levels. The training data set is prepared by extrapolating the charging voltage curves for the complete SOH range using initial 400 cycles of data. The trained artificial neural network (ANN) based on the feature vector and SOH values can be used in any battery management system (BMS) with a time complexity of only $O({n}^{4})$. Less than 1% mean absolute error (MAE) for the test cases has been achieved. The proposed method has a moderate training data requirement and does not need any knowledge of previous SOH, state of charge (SOC) vs. OCV relationship, and absolute SOC value.

## Introduction

Electric vehicles (EVs) have started replacing the traditional internal combustion (IC) engines slowly. Since sources of fossil fuels are gradually depleting, the demand for EVs is likely to increase many fold in the coming years. Another advantage of EVs is the reduction of greenhouse gases released in the atmosphere. The reliability of EV is one of the major challenges, and that is highly dependent on the condition of the battery under usage. Maximum possible distance the vehicle can be driven based on the current state of charge (SOC) of the battery, and whether the battery is safe enough to use are the two major concerns of the EV users. Both the queries can be addressed by accurate estimation of state of health (SOH) of the battery. Based on the literature, the common practice is to declare a battery as unsafe if the SOH falls below 80%. The amount of charge left in the battery is related to the amount of distance the EV can cover reliably. The amount of charge left is directly proportional to the product of the SOC and SOH. Therefore, online estimation of the SOH should be an integral part of any battery management system (BMS) for reliable operations of EVs. Other than the EVs, rechargeable batteries are used extensively in almost all the smart phones. The estimation of SOH for smart phone’s batteries are also important for device safety, reliability, and improved user experience. However, online estimation of the SOH is a challenging task considering the random usage pattern of the EVs, smart phones and most of the other battery operated systems. The charging and discharging of the batteries are normally partial and random in nature. The online SOH algorithm should be able to estimate the SOH accurately using the random partial charge-discharge data. Researchers have been working on this problem for more than a decade. Researchers are estimating the SOH by tracking various battery parameters like the remaining charge storing capacity, remaining energy storage capacity, increase in internal resistance, change in constant voltage (CV) charging current, etc. The SOH estimation methods can be divided into various subgroups such as data driven, electrochemical model based, electrical equivalent circuit based, etc.

Data driven methods are gaining popularity^1^ because they do not need detailed battery parameters to estimate SOH. An interesting data driven technique is reported in^2^, where the charging voltage curves are modelled with Gaussian process regression (GPR). Extended Kalman filter (EKF) is applied to estimate the constant current (CC) charging time, and battery capacity is estimated by Coulomb counting. This method is charging rate dependent and the estimated capacity is different from the 0.2 C rate capacity, which is followed in practice. Another method reported in^3^, applies GPR model to estimate SOH using the features from the estimated incremental capacity (IC) curve. IC curves are used in several other methods for SOH estimation^4,5^, since the peaks of the IC curves shift with ageing. In^4^, a linear regression fit is evaluated between the features of IC curve and the SOH for a training cell data, and estimated the SOH of other test cells using the relationship. An empirical model of OCV is developed in^5^, and used to estimate the SOH using IC analysis. The CV charging current changes with ageing. Several innovative methods extract various features from the CV phase current and relate that to the SOH of the battery^6,7^. SOH is estimated using the constant voltage (CV) charging capacity by applying an integrated quantum particle swarm optimization based support vector regression estimation framework in^6^. In^7^, the time constant of the CV time charging current is used to estimate SOH. CC completion time and times to complete various predefined segments of CV are used to train a random forest (RF) model to estimate SOH in^8^. If the battery is always charged at a fixed CC rate then, the current and voltage data between two fixed points during CC charging are used for SOH estimation^9,10^. The Coulomb counting value between the two fixed voltage points during the CC charging is directly used as the battery health index in^10^. Whereas, squared sum of voltage and charging time are also used along with the Coulomb counting as features to estimate SOH in^9^. Support vector machine (SVM) is used to map the feature space and the target SOH values. Another innovative data driven method is report in^11^, where a dynamically driven recurrent network (DDRN) with nonlinear autoregressive architecture and exogenous inputs is trained with the battery current, voltage and previous SOH values to estimate the current SOH. SOH is also estimated as the increase in the internal resistance of the battery. Internal resistance is estimated using various methods such as unscented KF (UKF)^12^, adaptive observer^13^, etc. An interesting approach is followed in^14^ to estimate the 10s discharge pulse resistance. First, an autoregressive moving average (ARMA) model is fitted between the recorded battery current and voltage data. The 10s discharge pulse resistance is estimated using the ARMA model. In^15^, the internal resistance along with the Coulomb count and cycle count are used to develop a numerical model for SOH estimation. A novel approach of estimating SOH as the ratio of the current energy storing capacity of the battery to its initial condition using the complete discharge data is reported in^16^.

Other than the data driven methods, the battery electrochemical model^17–19^ and the electrical equivalent circuit model (ECM)^20,21^ are also used extensively for SOH estimation. In^17^, EKF is applied on the single particle model of Li-ion battery to estimate the cyclable lithium, and from that SOH is derived. In^18^, first a reduced order electrochemical model is developed which includes the side reactions. The SOH is then estimated from the equilibrium potentials of the electrodes. ECM is used to coestimate SOC and SOH by applying EKF in^20^. The model parameters are estimated using the recursive least square (RLS) method from the current and voltage data. In^21^, a fractional-order battery equivalent circuit is developed and the parameters are optimized using a metaheuristics algorithm. Then, a fractional order KF is applied to estimate the SOC and SOH.

There are few novel methods reported in the literature where some kind of special arrangements are made to estimate the SOH^22–24^. In^22^, the response of an AC excitation current is processed using the nonlinear frequency response analysis (NFRA) technique to estimate the capacity fade due to the loss of active material. In^23^, the battery is charged with pulse charging technique and the electrical equivalent circuit parameters are estimated using two ANNs for lower and upper frequency ranges. The change in the circuit parameters indicates the battery degradation. Resting period battery voltage after the complete charging is used to estimate SOH in^24^.

SOH of the battery due to calendar aging is estimated using a feedforward artificial neural network (ANN) in^25^ using storage time, storage temperature and storage condition (fully-discharged or fully-charged) as inputs. In^26^, the calendar ageing is estimated using a portion of the voltage curve during C/5 CC charging.

Detailed reviews about the SOH estimation techniques are available in^27,28^. Other than the SOH estimation techniques, researchers are also studied various aspects of the battery degradation by modelling^29^. The effects of operating temperature and the current rate on the EV batteries are studied by modelling in^30^. Different types of battery models (electrochemical, semi-empirical, and empirical) for degradation study are compared in^31^. In^32^, ageing cost of the battery is derived by semi-empirical LiB degradation modelling for the power grid storage application.

Though the literature is rich with several SOH estimation algorithms, some practical issues still remained unaddressed. The following areas in the existing literatures are needed to be solved for the online estimation of SOH in any BMS.

1. Some algorithms can only estimate SOH for the same range of values for which the training has been performed. Therefore, a lot of training data and time would require to enable such algorithms for the estimate of low SOH values^3,4,9^.
2. Complete charging or discharging battery data is needed for few cases^16,24^. Under practical uses, the batteries are normally charged and discharged partially, and the extent of charging or discharging is quite random. Therefore, complete charging or discharging data may not always be available.
3. Special kinds of probing signals are injected for SOH estimation, which may not be feasible when the battery is in use^22,23^.
4. In some algorithms, cycle number is used as a parameter to estimate SOH^15^. However, we may not have a proper cycle number when most of the times the batteries are charged and discharged partially.
5. Few reported methods use battery model and the accuracy of the estimated SOH is dependent on the accuracy of the model parameters^17,18^. The model parameters also change with ageing.
6. There are few methods reported in literature that are only suitable for offline estimation of the SOH^27^.

The challenge of estimating SOH using the partial charging data is addressed by designing a novel feature vector consisting of differences in the derived voltage values and average battery temperature. We have used approximately 15 min of charging data to generate the feature vector for each partial cycle. The feature vectors are generated corresponding to different levels of SOH between 100% to 80% for training purpose. We have applied k-nearest neighbour (kNN), linear regression, SVM regression, random forest (RF), and ANN to fit a model between the feature vectors and the target SOH values. ANN is found to show better accuracy for the problem in hand. Therefore, we have showed only the ANN results in this paper. First, an ANN model is trained using the training dataset. Now the challenge is to generate the training data for the SOH range of 100% to 80% in a shorter span of time. Cycling of the cells for the training range of SOH may take several months. The battery manufacturers generally charge and discharge few batteries from a particular batch for 300 ∼ 400 cycles (approx.) before the deployment^33,34^. Therefore, we have devised a unique approach to extrapolate the charging voltage curves for the complete training SOH range using the initial 400 cycles of charge-discharge battery data. The 400 cycles of battery data are generated in less than 45days time in the laboratory. The trained ANN model is then used to estimate the SOH of other test batteries. We have achieved ≤1% mean absolute error (MAE) in SOH estimation for the test data. The proposed method can estimate SOH of cells with capacities different from the training cells, but with similar electrochemistry. The novel features of the proposed method are as follows.

1. Partial charging data of 10–20 min duration is sufficient to estimate the SOH.
2. A novel differential voltage based feature vector is designed to estimate the SOH.
3. Only initial 400 cycles (∼45 days) of charge-discharge cycle data is needed for training.
4. Training and testing batteries can have different capacities. In our experiments, we have used 3.0 Ah battery data for training and tested on 3.5 Ah battery of similar electro-chemistry.

A comparison between the existing methods and the proposed method is given in Table 1

**Table 1:** Novelty of the proposed method.

Existing methods	Proposed method
Large training data	Only initial 400 cycles (∼45 days) needed for training
Complete charging/discharging data	Partial charging data 10–20 min
Special kinds of probing signals	Existing partial charge data
Cycle numbers required	Differential voltage based feature vector
Battery models with model parameters	One time training
Offline estimation	Online on the device

This paper is organized as follows. The Results section discusses the feature set used for SOH estimation, details of training and testing, followed by a short Discussion. The Methods section includes details of experimental data generation and extrapolation of voltage curves.

## Results

### Design of feature vector

**Figure 1:**
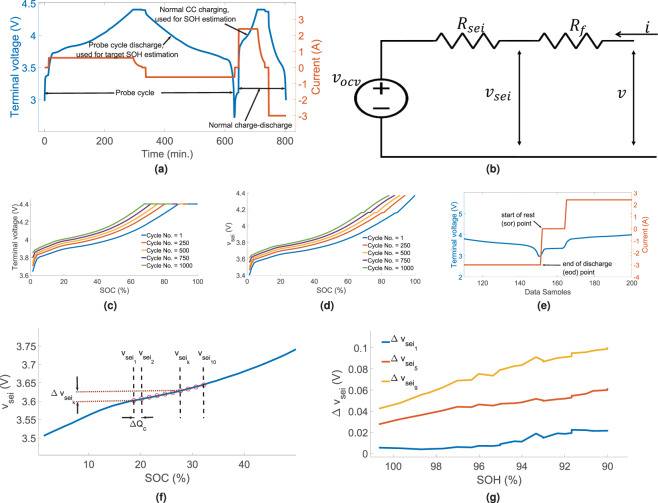
(**a**) Sample Charge-Discharge cycle current and voltage plot, Probe cycle and normal CC-CV. (**b**) Battery electrical equivalent circuit. Battery voltage vs. SOC plots at different ageing stages, (**c**) Terminal Voltage and (**d**) ${v}_{sei}$, (**e**) Voltage and current curves during discharge to charge transition, (**f**) Selection of $\Delta {v}_{sei}$ points, (**g**) $\Delta {v}_{se{i}_{k}}$ vs. SOH plot.

A simplified battery model is assumed consisting of two resistive elements (${R}_{f}$ and $\Delta {R}_{sei}$) connected in series with the OCV (${v}_{ocv}$) as shown in Fig. 1b. ${R}_{f}$ is the fixed internal resistance of the battery which does not change with aging. $\Delta {R}_{sei}$ is the increase in the internal resistance due to the increase in the thickness of the solid-electrolyte interface (SEI) layer. Therefore, the battery internal resistace, ${R}_{i}={R}_{f}+\Delta {R}_{sei}$. Therefore, for the first cycle, ${R}_{i}={R}_{f}$. Figure 1c shows the battery terminal voltage ($V$) vs. SOC and ${v}_{sei}$ vs. SOC plots corresponding to different cycle numbers during charging. The plots in Fig. 1c,d illustrates how the terminal voltage and the ${v}_{sei}$ curves change with ageing. The data for the plots are generated from a Type-1 battery cycled by 0.8 C charging and 1.0 C discharging at 45 °*C*. The SOC for the plot is estimated as

1 $$SOC(k)=SOC(k-\mathrm{1)}+\frac{i(k){T}_{s}}{{C}_{max}}$$

where $i(k)$ is the $K$-th instant current, ${T}_{s}$ is the sampling time, and ${C}_{max}$ is the rated capacity of the battery. The initial SOC is assumed to be zero. ${v}_{sei}$ is estimated from the battery current ($i(k)$) and voltage ($v(k)$) data as,

2 $${v}_{sei}(k)=v(k)-{R}_{f}i(k)$$

${R}_{f}$ is estimated from the first charge-discharge cycle data as follows, since for the first cycle, ${R}_{i}={R}_{f}$.

3 $${R}_{f}=\frac{v(eod)-v(sor)}{i(eod)}$$

where $eod$ means end of discharge point as indicated in Fig. 1e. $sor$ means start of rest point, just after the end of discharging phase. During resting, $i(k)=0$. The slow charge-discharge cycle at 0.2 C rate, which is also referred to as the probing cycle, is used for target SOH estimation. The SOH is estimated as,

4 $$capacity(n)=-{T}_{s}\sum _{k\in discharging}{i}_{b}(k)$$

5 $$SOH(n)=\frac{capacity(n)}{{C}_{max}}\times \mathrm{100 \% }$$

where ${i}_{b}(k)$ is the probe cycle current during discharge. *n* denotes the current cycle number. The summation in (4) is taken for the complete discharge period. Careful inspection of Fig. 1c,d reveals that the voltage vs. SOC curves shrink with decrease in SOH. SOH decreases with the increase in cycle number. The shrinkage in the horizontal axis happens due to the active material loss as well as loss of Lithium inventory, and the shrinkage in the vertical axis happens due to the increase in internal resistance. Loss of active material results in permanent capacity loss of the battery. The internal resistance increases with ageing mostly due to the increase in SEI layer on the anode surface. There is a strong correlation between the internal resistance increase due to the growth of anode SEI layer and the permanent capacity loss^35^. This relationship is discussed and applied to extrapolate the voltage curves for different SOH values in the Methods section. We have used ${v}_{sei}$ instead of the terminal voltage ($v$) for SOH estimation to reduce the effects of different CC charging rates on the estimation process. To generate the feature vector, first a fixed ${v}_{sei}$ value (${v}_{se{i}_{0}}$) is selected on the ${v}_{sei}$ curve during charging. The selection of this fixed value could depend on the specific application, say 3.7 V if the battery is charged and discharged between 20% to 60% of SOC. The peak of an Incremental Capacity curve can also be taken as the initial point provided it is available in the partial charging data^36^. The next ${v}_{sei}$ point is selected from the charging ${v}_{sei}$ curve in such a way that the Coulomb count ($\Delta {Q}_{c}$) between those two points is as follows

6 $$\Delta {Q}_{c}\,=\,\sum i(k){T}_{s}\,\mathrm{=}\,\mathrm{1.5 \% }\,{\rm{of}}\,{C}_{max}$$

The same process is repeated to collect total 10 ${v}_{sei}$ points from the charging ${v}_{sei}$ curve with $\Delta {Q}_{c}$ gap between any two consecutive points as shown in Fig. 1f. The feature vector is constructed as

7 $$x=[\begin{array}{ccccc}\Delta {v}_{se{i}_{1}} & \Delta {v}_{se{i}_{2}} & \ldots  & \Delta {v}_{se{i}_{9}} & {T}_{avg}\end{array}]$$

where ${T}_{avg}$ is the average battery temperature during charging, and $\Delta {v}_{se{i}_{k}}$ is evaluated as

8 $$\Delta {v}_{se{i}_{k}}={v}_{se{i}_{k}}-{v}_{se{i}_{k-1}},\,k\mathrm{=1,2,}\ldots \mathrm{,9}$$

Figure 1g shows the $\Delta {v}_{se{i}_{k}}$ vs. SOH plots for three different values of $k$. Since the relationship between the $\Delta {v}_{se{i}_{k}}$ and SOH is complex, we have used an ANN framework to learn the relationship between the feature vectors and the target SOH values.

**Figure 2:**
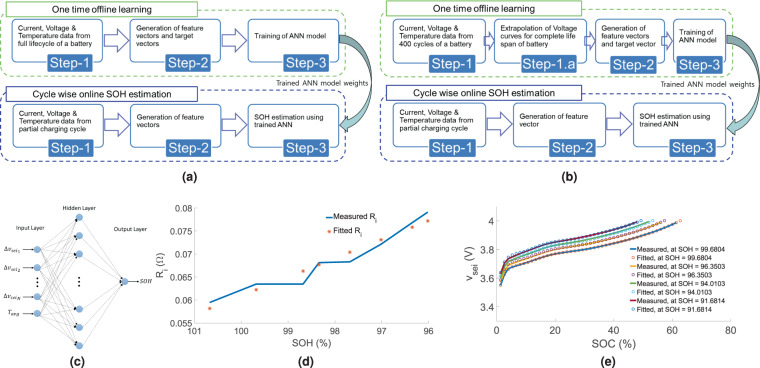
Flow diagram of the proposed method when complete lifespan of battery data (**a**) available, (**b**) not available. (**c**) Schematic diagram of ANN for SOH estimation. (**d**) Internal resistance vs SOH curve, measured and fitted over initial 400 cycles. The correlation co-efficient between the measured and the fitted values is 0.9665. (**e**) ${v}_{sei}$ vs. $SOC$ curves at different SOH, measured and fitted. The correlation co-efficients between the measured and the fitted values, for decreasing SOH have been found to be 0.9999, 0.9998, 0.9998 and 0.9997.

Figure 2a shows the block diagram of the proposed methodology when the charge-discharge cycling data for the complete lifespan (100–80%) of the battery is available to train the algorithm. Generation of charge-discharge data for the complete lifespan of a battery is a time-consuming activity. Under practical situations, only few batteries from a particular batch are cycled for 300 ∼ 400 cycles (approx.) before deployment. The challenge is to generate feature vectors for the complete lifespan of the battery from the initial 400 cycles of charge-discharge data. The proposed technique of extrapolating the charging voltage curves using the initial 400 cycles is indicated as a processing block in Fig. 2b. One time offline learning is followed by Cycle wise online SOH estimation to estimate SOH systematically. Training of the ANN using the feature vectors and target values. The ANN network used to generate the results in this paper is having only one hidden layer. A schematic diagram of the ANN is shown in Fig. 2c. The ANN model has 10 input nodes, one output node, and one hidden layer with 100 nodes.

### Offline learning

**Figure 3:**
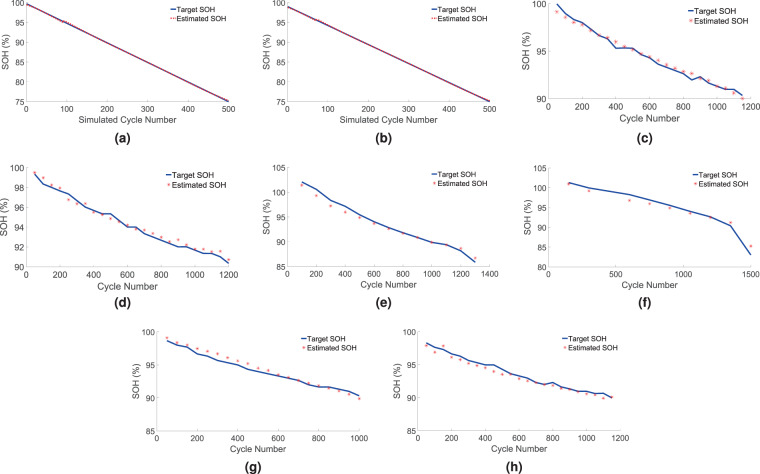
Training on extrapolated battery degradation data, Chamber temperature (**a**) 45 °*C* and (**b**) 25 °*C*. SOH estimation (target and estimated), SOH vs. Cycle number plots for two Type-1 batteries, CC charging rate 0.8*C*, Chamber temperature (**c**) 45 °*C* and (**d**) 25 °*C*. SOH vs. Cycle number plots for two Type-1 batteries, Chamber temperature 45 °*C*, CC charging rates (**e**) 1.2*C* and (**f**) 1.0*C*. SOH vs. Cycle number plots for two Type-2 batteries, CC charging rate 0.8*C*, Chamber temperature (**g**) 45 °*C* and (**h**) 25 °*C*.

For the one time offline learning, two batteries of Type-1 are selected, as mentioned in Table 3, one of which was cycled at 45 °*C* and the other one at 25 °*C*. Using the information from the initial 400 cycles of charging and discharging data the voltage curves have been extrapolated from $\mathrm{100 \% }$ to $\mathrm{75 \% }$ in $500$ equal steps. The feature vectors are extracted from the extrapolated voltage curves. The feature vectors and the target SOH values are used for training an ANN model. Logistic activation function is used for the nodes. After few trials, we have selected the mentioned architecture keeping a balance between the amount of computation needed and the required accuracy. ANN has been implemented using the Python package scikit-learn. Figure 3a,b shows the target SOH and the estimated SOH for the training data at 45 °*C* and 25 °*C* respectively. As expected, they are very close to each other since ANN model has been trained with the same data. SOH has been plotted with respect to the cycle numbers in all the figures, but the cycle number information has not been used anywhere to estimate the SOH.

### SOH estimation

The trained model is then used for SOH estimation of other test batteries using partial charging data. The test batteries are different from the training batteries. Figure 3c,d show the SOH (target and estimated) vs. cycle number plots for the two Type-1 batteries. The two Type-1 batteries under test were cycled at $0.8$ C CC charging rate and the chamber temperature was 45 °*C* and 25 °*C* respectively. The proposed method tracks the SOH accurately for the testing range of ∼100% to ∼90% SOH. Therefore, the trained model estimates SOH precisely beyond the range of the training data which was the initial $400$ cycles (∼100% to ∼96% SOH) from two different batteries.

To test the performance at lower range of SOH ($\mathrm{ < 90 \% }$), two Type-1 batteries were cycled at higher CC charging rates of $1.2C$ and $1.0C$ respectively at 45 °*C* chamber temperature. Probe cycling was performed for every $125$ normal charge-discharge cycles. The lowest SOH reached approximately $\mathrm{85 \% }$. The SOH (target and estimated) vs. cycle number plots are shown in Fig. 3e,f for the two lower SOH test batteries. Both the cases, the proposed method tracks the SOH till the end point accurately. Another noteworthy advantage is that, the testing CC rates are different from the training rates and still the proposed algorithm achieved high accuracy in SOH estimation.

Figure 3g,h show the SOH (target and estimated) vs. cycle number plots for two Type-2 batteries cycled at two different temperatures (Table 3). Even though, the training is performed using only Type-1 batteries of $3.0$ Ah capacity, the trained model is capable of estimating the SOH of Type-2 batteries with 3.5. Ah capacity. This advantage of the proposed method comes from the two reasons such as, both the Type-1 and Type-2 batteries are having same electrochemistry and the SOH is always normalized with the maximum capacity of the battery under test. We have tested total 16 batteries (Table 3) to prove the accuracy and robustness of our proposed method. Three metrics, mean absolute error (MAE), standard deviation (SD) of the absolute error and maximum absolute error (MaxE), are used for performance evaluation. Absolute error is evaluate as

9 $$AE=|\frac{SO{H}_{t}-SO{H}_{e}}{SO{H}_{t}}|$$

**Figure 4:**
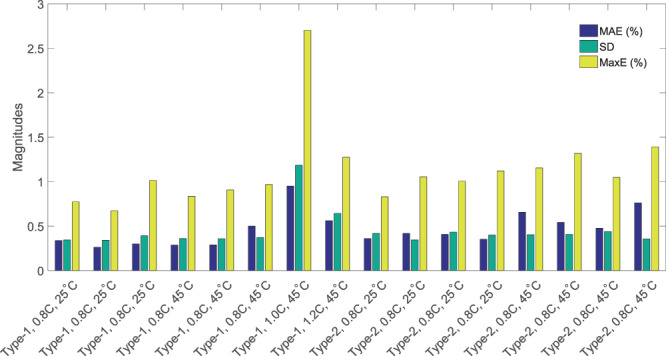
Bar plot of MAE, SD, and MaxE for 16 test batteries.

Where $\mathrm{|.|}$ is the absolute value operator. $SO{H}_{t}$ is the target SOH, evaluated from the probing cycle data. $SO{H}_{e}$ is the estimated SOH from the proposed method. The statistical performance for all the test cases is summarized in Fig. 4. Figure 4 shows the bar plots for MAE, SD and MaxE. It is observed from the plots that the MAE is always below $\mathrm{1 \% }$ and SD is also below $0.7$ except for one case. The MaxE is also always below $\mathrm{1.5 \% }$ except for one case. Therefore, we can state that the proposed method is having high accuracy and robustness. The performance of the testing stage can be found to be $O({n}_{layer}\mathrm{\ .\ }{n}_{neurons}^{3})$ assuming a neural network with ${n}_{layer}$ layers, each having ${n}_{neuron}$ neurons.

## Discussion

An online solution is developed to solve the practical problem of estimating SOH using the partial charging data. Approximately 15 min of normal usage charging data is sufficient to estimate the SOH. The proposed solution collects current, voltage, and temperature data from the existing BMS and can be integrated easily with the BMS code base. To train the algorithm, 400 cycles of charge-discharge data are needed. Therefore, for a new battery chemistry, the SOH estimation algorithm can be ready within 45 days (approx.). If the battery capacity changes without any change in the electrochemistry then no new training is needed. We have tested the proposed method under different test conditions and found to be accurate ($MAE < \mathrm{1 \% }$) and robust. Another practical advantage is that, only the first cycle internal resistance is needed to estimate the SOH for the rest of the life. As a future scope of this work, we are working on reducing the training data requirement further from 400 cycles of initial charge-discharge data. Since there is no restriction in the methodology on charging rates, higher C-rates will also be explored. With larger datasets, a deep neural network can be used to directly fit the data. Further, a more sophisticated model compared to the OCV-resistance relationship used here can also be used^37^. The proposed method is equally applicable to EVs, smart phones, and other battery operated systems for battery SOH estimation.

## Methods

### Experimental data generation

Two types of batteries with Lithium Cobalt Oxide (LCO) cathode and graphite anode were used for experimentation. Both the battery types had pouch cell geometry with nominal voltage 3.85–4.4 V. 10 batteries of Type-1 with max capacity 3.0 Ah and 8 batteries of Type-2 with max capacity of 3.5 Ah were used.

Total 18 batteries from the two types were used for training and testing data generation. Batteries were cycled at two different temperatures, 45 °*C* and 25 °*C*, by keeping them inside the thermal chambers. Table 2 shows the charging and discharging protocols followed for cycling the batteries. 0.2 C rate discharging data were used to estimate the target SOH values. The 0.8 C data was sampled at 1 min and 1.2 C data at 10 sec intervals. Using a cubic polynomial all data was upscaled to a 1 sec sampling. The details of the training and testing conditions are provided in Table 3. One normal charge-discharge cycle and one probe cycle profile of current and voltage are shown in Fig. 1a.

**Table 2:** Charging and Discharging Protocol.

Step	Protocol Description
1	Constant current (CC) charging at 0.8 C/1.0 C/1.2 C rate
2	Constant voltage (CV) charging at 4.4 V
3	Constant current discharge at 1.0 C/1.2 C rate
4	After each 50/125 cycles, probe cycle with CC-CV charge and CC discharge at 0.2 C rate

**Table 3:** Training and Testing Split.

Sl.No.	Battery Type	No. of battery used	CC Charging rate	Chamber Temp. (°*C*)	Used for
1	Type-1	1	0.8C	45	Training
2	Type-1	1	0.8C	25	Training
3	Type-1	3	0.8C	45	Testing
4	Type-1	3	0.8C	25	Testing
5	Type-1	1	1.0C	45	Testing
6	Type-1	1	1.2C	45	Testing
7	Type-2	4	0.8C	45	Testing
8	Type-2	4	0.8C	25	Testing

### Extrapolation of voltage curves

The extrapolation process of the charging voltage curves for the complete lifespan of the battery using the information from the initial 400 cycles of charge-discharge data is discussed. Simplified analytical expressions are provided in^35^ for capacity loss (${Q}_{Loss}$ (10)) and the resistance of the SEI layer (${R}_{sei}$ (11)).

10 $${Q}_{Loss}={a}_{s}^{n}{i}_{\mathrm{0,}sei}{A}_{n}{L}_{n}t\left(1+{\left[\frac{{I}_{rms}}{{I}_{a}}\right]}^{2}\right)exp\left[-\frac{{\alpha }_{n}F}{RT}{\bar{\eta }}_{sei}\right]$$

11 $${R}_{sei}={R}_{sei\mathrm{,0}}+\frac{{\delta }_{sei}}{{\kappa }_{sei}}$$

12 $${\delta }_{sei}=\frac{{M}_{sei}{i}_{\mathrm{0,}sei}t}{{\rho }_{sei}F{A}_{n}}\left(1+{\left[\frac{{I}_{rms}}{{I}_{a}}\right]}^{2}\right)exp\left[-\frac{{\alpha }_{n}F}{RT}{\bar{\eta }}_{sei}\right]$$

The descriptions of the symbols used in (10)-(12) are provided in Table 4. Combining (12) and (10) we get,

13 $${\delta }_{sei}=\frac{{M}_{sei}}{{\rho }_{sei}F{a}_{s}^{n}{A}_{n}^{2}{L}_{n}}{Q}_{Loss}$$

**Table 4:** Symbols.

Sl. No.	Symbol	Name
1	${a}_{s}^{n}$	specific interfacial area of anode, $c{m}^{-1}$
2	${i}_{\mathrm{0,}sei}$	initial side reaction exchange current density, $Ac{m}^{-2}$
3	${A}_{n}$	area of anode, $c{m}^{-2}$
4	${L}_{n}$	thickness of anode, $cm$
5	$T$	cycle time, $s$
6	${I}_{rms}$	RMS value of the input current, $A$
7	${I}_{a}$	$\frac{\sqrt{2}RT}{{\alpha }_{n}F{C}_{1}}$, $A$
8	$R$	universal gas constant, $Jmo{l}^{-1}{K}^{-1}$
9	$T$	temperature, $K$
10	${\alpha }_{n}$, ${\alpha }_{p}$	charge transfer coefficient of anode and cathode respectively
11	$F$	Faraday constant, $Cmo{l}^{-1}$
12	${C}_{1}$	$\frac{{R}_{ct}}{{a}_{s}^{n}{A}_{n}{L}_{n}}+\frac{\partial {U}_{n}}{\partial {C}_{s,e}}\frac{{g}_{2}}{{A}_{n}{L}_{n}}$
13	${R}_{ct}$	$\frac{RT}{{i}_{0}^{n}({\alpha }_{p}+{\alpha }_{n})F}$
14	${i}_{0}^{n}$	exchange current density of anode, $Ac{m}^{-2}$
15	${U}_{n}$	open circuit potential of anode, $V$
16	${C}_{s,e}$	solid particle Li concentration in both electrode
17	${g}_{2}$	$\frac{-{R}_{s}^{n}}{5{D}_{s}^{n}F{a}_{s}^{n}}$
18	${R}_{s}^{n}$	particle radius of anode, $cm$
19	${D}_{s}^{n}$	solid phase Li diffusion coefficient of anode, $c{m}^{2}{s}^{-1}$
20	${\bar{\eta }}_{sei}$	average SEI overpotential at equilibrium, $V$
21	${R}_{sei\mathrm{,0}}$	initial SEI film resistance, $\Omega$
22	${M}_{sei}$	SEI layer Molar mass, $kgmo{l}^{-1}$
23	${\rho }_{sei}$	SEI layer density, $kgc{m}^{-3}$

Combining (13) and (11) we get,

14 $${R}_{sei}={R}_{sei\mathrm{,0}}+\frac{{M}_{sei}}{{\kappa }_{sei}{\rho }_{sei}F{a}_{s}^{n}{A}_{n}^{2}{L}_{n}}{Q}_{Loss}$$

The increase in the SEI layer resistance ($\Delta {R}_{sei}$) from cycle number ${j}_{1}$ to ${j}_{2}$ (${j}_{2} > {j}_{1}$) is derived from (14) as follows,

15 $$\Delta {R}_{sei}({j}_{2},{j}_{1})={P}_{c}\Delta {Q}_{Loss}({j}_{2},{j}_{1})$$

16 $$where,\,{P}_{c}=\frac{{M}_{sei}}{{\kappa }_{sei}{\rho }_{sei}F{a}_{s}^{n}{A}_{n}^{2}{L}_{n}}$$

17 $$\Delta {R}_{sei}({j}_{2},{j}_{1})={R}_{sei}({j}_{2})-{R}_{sei}({j}_{1})$$

18 $$\Delta {Q}_{Loss}({j}_{2},{j}_{1})={Q}_{Loss}({j}_{2})-{Q}_{Loss}({j}_{1})$$

The relationship between $\Delta {R}_{sei}$ and $\Delta {Q}_{loss}$ (15) contains several battery parameters and getting those parameter values may not be feasible for every commercial batteries. Therefore, we have assumed ${P}_{c}$ as a single parameter and estimated ${\hat{P}}_{c}$ using the initial 400cycles of data. $(\hat{.})$ indicates the estimated quantity. First, the capacity (4) and the SOH (5) are evaluated using the probing cycles till 400th cycle. The internal resistance (3) is evaluated using the normal charge-discharge cycles just before the probing cycles. ${\hat{P}}_{c}$ is evaluated using the least square method as follows.

19 $${\hat{P}}_{c}=({\boldsymbol{\Delta }}{{{\bf{Q}}}_{{\bf{l}}{\bf{o}}{\bf{s}}{\bf{s}}}}^{T}{\boldsymbol{\Delta }}{{\bf{Q}}}_{{\bf{l}}{\bf{o}}{\bf{s}}{\bf{s}}}{)}^{-1}{\boldsymbol{\Delta }}{{{\bf{Q}}}_{{\bf{l}}{\bf{o}}{\bf{s}}{\bf{s}}}}^{T}{\boldsymbol{\Delta }}{{\bf{R}}}_{{\bf{s}}{\bf{e}}{\bf{i}}}$$

where, ${\mathrm{(.)}}^{T}$ represents transpose.

20 $${\boldsymbol{\Delta }}{{\bf{Q}}}_{{\bf{L}}{\bf{o}}{\bf{s}}{\bf{s}}}=[\Delta {Q}_{Loss}\mathrm{(50,0)}\,\Delta {Q}_{Loss}\mathrm{(100,0)}\cdots \Delta {Q}_{Loss}{\mathrm{(400,0)]}}^{T}$$

21 $${\boldsymbol{\Delta }}{{\bf{R}}}_{{\bf{s}}{\bf{e}}{\bf{i}}}=[\Delta {R}_{sei}\mathrm{(50,0)}\,\Delta {R}_{sei}\mathrm{(100,0)}\,\cdots \,\Delta {R}_{sei}{\mathrm{(400,0)]}}^{T}$$

22 $$\Delta {R}_{sei}\mathrm{(50,0)}={R}_{i}\mathrm{(50)}-{R}_{i}\mathrm{(0)}$$

23 $${R}_{i}\mathrm{(50)=}{R}_{f}+\Delta {R}_{sei}\mathrm{(50,0)}$$

24 $${R}_{f}={R}_{sei}\mathrm{(0)}+other\,fixed\,resistances$$

Figure 2d shows the internal resistance vs. SOH curves for the initial 400 cycles. The solid blue line represents the ${R}_{i}$ which is directly evaluated from the data using (3) and the red stars represent the fitted ${R}_{i}$ using (15), (19), and (23) for the same SOH. The extrapolation process of voltage curves (${v}_{sei}$) is explained stepwise as follows.

**Step 1**: Evaluation of OCV (${v}_{ocv}$) and SOC from the first normal charging cycle current and voltage data. OCV is evaluated using the following voltage relationships for the simplified battery model (Fig. 1b).

25 $${v}_{ocv}(k)={v}_{sei}(k)-\Delta {R}_{sei}i(k)$$

26 $${v}_{sei}(k)=v(k)-{R}_{f}i(k)$$

For the first cycle (SOH 100%), $\Delta {R}_{sei}=\Delta {R}_{sei}\mathrm{(0,0)}=0$ and $\Delta {Q}_{loss}\mathrm{(0,0)}=0$. Therefore, ${v}_{sei}(k)={v}_{ocv}(k)$. SOC for the first cycle is estimated using (1). The initial SOC is taken to be zero. The advantage of the proposed voltage extrapolation method is that it is independent to the choice of the initial SOC.

**Step 2**: Fitting of a polynomial of $N$-th order between the ${v}_{ocv}$ and $SOC$.

27 $${v}_{ocv}(k)={a}_{0}+{a}_{1}SOC(k)+{a}_{2}SO{C}^{2}(k)+\ldots +{a}_{N}SO{C}^{N}(k)$$

where ${a}_{i}$ s are the coefficients of the polynomial.

**Step 3**: Evaluation of the ${v}_{ocv}$ vs. $SOC$ curve at $x \%$ SOH. The ${v}_{ocv}$ and $SOC$ curve from the first cycle is shrunk in the horizontal direction due to the SOH reduction as

28 $${v}_{ocv}(k,at\,x \% \,SOH)={a}_{0}+{a}_{1}\left(\frac{xSOC(k)}{100}\right)+{a}_{2}{\left(\frac{xSOC(k)}{100}\right)}^{2}+\ldots +{a}_{N}{\left(\frac{xSOC(k)}{100}\right)}^{N}$$

**Step 4**: Evaluation of the ${v}_{sei}$ vs. $SOC$ curve at $x \%$ SOH.

29 $${v}_{sei}(k,at\,x \% \,SOH)={v}_{ocv}(k,at\,x \% \,SOH)+\Delta {R}_{sei}(at\,x \% \,SOH)i(k)$$

where

30 $$\Delta {R}_{sei}(at\,x \% \,SOH)={\hat{P}}_{c}\Delta {Q}_{loss}(at\,x \% \,SOH)$$

31 $$\Delta {Q}_{loss}(at\,x \% \,SOH)=\frac{\mathrm{(100}-x)}{100}{C}_{max}$$

To illustrate the effectiveness of the proposed method, we have shown the ${v}_{sei}$ vs. $SOC$ plots in Fig. 2e at four different SOH levels. The solid lines represent experimental data and the bubble plots are the extrapolated ${v}_{sei}$ vs. $SOC$ curves. The plots show that the measured curves and the extrapolated curves are in close proximity ($\mathrm{ < 1 \% }$ mean square error).

## References

[1] Tagade P (2020). Deep gaussian process regression for lithium-ion battery health prognosis and degradation mode diagnosis. J. Power Sources.

[2] Zhou, D., Yin, H., Xie, W., Fu, P. & Lu, W. Research on online capacity estimation of power battery based on ekf-gpr model. *J. Chem*. **2019** (2019).

[3] Li, X. & Wang, Z. State of health estimation for lithium-ion battery by combing incremental capacity analysis with gaussian process regression. *arXiv preprint arXiv:1903.07672* (2019).

[4] Li Y (2018). A quick on-line state of health estimation method for li-ion battery with incremental capacity curves processed by gaussian filter. J. Power Sources.

[5] Weng C, Sun J, Peng H (2014). A unified open-circuit-voltage model of lithium-ion batteries for state-of-charge estimation and state-of-health monitoring. J. power Sources.

[6] Wang Z, Zeng S, Guo J, Qin T (2018). Remaining capacity estimation of lithium-ion batteries based on the constant voltage charging profile. PloS one.

[7] Yang J, Xia B, Huang W, Fu Y, Mi C (2018). Online state-of-health estimation for lithium-ion batteries using constantvoltage charging current analysis. Appl. energy.

[8] Chen, Z., Sun, M., Shu, X., Shen, J. & Xiao, R. On-board state of health estimation for lithium-ion batteries based on random forest. In *2018 IEEE International Conference on Industrial Technology (ICIT)*, 1754–1759 (IEEE, 2018).

[9] Chen Z, Sun M, Shu X, Xiao R, Shen J (2018). Online state of health estimation for lithium-ion batteries based on support vector machine. Appl. Sci..

[10] Xiong R (2018). Lithium-ion battery health prognosis based on a real battery management system used in electric vehicles. IEEE Transactions on Veh. Technol..

[11] Chaoui H, Ibe-Ekeocha CC (2017). State of charge and state of health estimation for lithium batteries using recurrent neural networks. IEEE Transactions on vehicular technology.

[12] Qiuting W, Yinzhu J, Yunhao L (2015). State of health estimation for lithium-ion battery based on d-ukf. Int. J. Hybrid Inf. Technol.

[13] Gholizadeh M, Salmasi FR (2013). Estimation of state of charge, unknown nonlinearities, and state of health of a lithium-ion battery based on a comprehensive unobservable model. IEEE Transactions on Ind. Electron..

[14] Giordano G, Klass V, Behm M, Lindbergh G, Sjöberg J (2018). Model-based lithium-ion battery resistance estimation from electric vehicle operating data. IEEE Transactions on Veh. Technol..

[15] Yu J (2017). Indirect state-of-health estimation for lithium-ion batteries under randomized use. Energies.

[16] Diao W, Jiang J, Zhang C, Liang H, Pecht M (2017). Energy state of health estimation for battery packs based on the degradation and inconsistency. Energy Procedia.

[17] Huang, M., Kumar, M., Yang, C. & Soderlund, A. Aging estimation of lithium-ion battery cell using an electrochemical model-based extended kalman filter. In *AIAA Scitech 2019 Forum*, 0785 (2019).

[18] Gao Y, Zhang X, Yang J, Guo B (2018). Estimation of state-of-charge and state-of-health for lithium-ion degraded battery considering side reactions. J. The Electrochem. Soc..

[19] Tagade P (2016). Bayesian calibration for electrochemical thermal model of lithium-ion cells. J. Power Sources.

[20] Shen P, Ouyang M, Lu L, Li J, Feng X (2017). The co-estimation of state of charge, state of health, and state of function for lithium-ion batteries in electric vehicles. IEEE Transactions on vehicular technology.

[21] Hu X, Yuan H, Zou C, Li Z, Zhang L (2018). Co-estimation of state of charge and state of health for lithium-ion batteries based on fractional-order calculus. IEEE Transactions on Veh. Technol..

[22] Harting N, Wolff N, Röder F, Krewer U (2019). State-of-health diagnosis of lithium-ion batteries using nonlinear frequency response analysis. J. The Electrochem. Soc..

[23] Bezha M, Gondo R, Nagaoka N (2019). An estimation model with generalization characteristics for the internal impedance of the rechargeable batteries by means of dual ann model. Energies.

[24] He, L., Kim, E., Shin, K. G., Meng, G. & He, T. Battery state-of-health estimation for mobile devices. In *2017 ACM/IEEE 8th International Conference on Cyber-Physical Systems (ICCPS)*, 51–60 (IEEE, 2017).

[25] Kashkooli AG, Fathiannasab H, Mao Z, Chen Z (2019). Application of artificial intelligence to state-of-charge and state-of-health estimation of calendar-aged lithium-ion pouch cells. J. The Electrochem. Soc..

[26] Stroe DI, Knap V, Schaltz E (2018). State-of-health estimation of lithium-ion batteries based on partial charging voltage profiles. Ecs Transactions.

[27] Sarmah SB (2019). A review of state of health estimation of energy storage systems: Challenges and possible solutions for futuristic applications of li-ion battery packs in electric vehicles. J. Electrochem. Energy Convers. Storage.

[28] Lipu MH (2018). A review of state of health and remaining useful life estimation methods for lithium-ion battery in electric vehicles: Challenges and recommendations. J. Clean. Prod..

[29] Hariharan, K. S., Tagade, P. & Ramachandran, S. *Mathematical Modeling of Lithium Batteries: From Electrochemical Models to State Estimator Algorithms* (Springer, 2017).

[30] Yang Z, Patil D, Fahimi B (2018). Electrothermal modeling of lithium-ion batteries for electric vehicles. IEEE Transactions on Veh. Technol..

[31] Jin X (2018). Applicability of available li-ion battery degradation models for system and control algorithm design. Control. Eng. Pract..

[32] Xu B, Oudalov A, Ulbig A, Andersson G, Kirschen DS (2018). Modeling of lithium-ion battery degradation for cell life assessment. IEEE Transactions on Smart Grid.

[33] Insights, M. T. R. Samsung’s quest to mitigate the battery challenge (2017).

[34] Mikolajczak, C. J., Hayes, T., Megerle, M. V. & Wu, M. A scientific methodology for investigation of a lithium ion battery failure. In *2007 IEEE International Conference on Portable Information Devices*, 1–6 (IEEE, 2007).

[35] Tanim TR, Rahn CD (2015). Aging formula for lithium ion batteries with solid electrolyte interphase layer growth. J. Power Sources.

[36] Tang X (2018). A fast estimation algorithm for lithium-ion battery state of health. J. Power Sources.

[37] Tang X (2019). A novel framework for lithium-ion battery modeling considering uncertainties of temperature and aging. Energy conversion management.

